# Primordial Germ Cell Development in the Poeciliid, *Gambusia holbrooki*, Reveals Shared Features Between Lecithotrophs and Matrotrophs

**DOI:** 10.3389/fcell.2022.793498

**Published:** 2022-03-01

**Authors:** Komeil Razmi, Jawahar G. Patil

**Affiliations:** Laboratory of Molecular Biology, Fisheries and Aquaculture Centre, Institute for Marine and Antarctic Studies, University of Tasmania, Taroona, TAS, Australia

**Keywords:** germline development, germ plasm, invasive species, evolutionary adaptation, viviparity

## Abstract

Metazoans exhibit two modes of primordial germ cell (PGC) specification that are interspersed across taxa. However, the evolutionary link between the two modes and the reproductive strategies of lecithotrophy and matrotrophy is poorly understood. As a first step to understand this, the spatio-temporal expression of teleostean germ plasm markers was investigated in *Gambusia holbrooki*, a poecilid with shared lecitho- and matrotrophy. A group of germ plasm components was detected in the ovum suggesting maternal inheritance mode of PGC specification. However, the strictly zygotic activation of *dnd-β* and *nanos1* occurred relatively early, reminiscent of models with induction mode (*e.g.,* mice). The PGC clustering, migration and colonisation patterns of *G. holbrooki* resembled those of zebrafish, medaka and mice at blastula, gastrula and somitogenesis, respectively—recapitulating features of advancing evolutionary nodes with progressive developmental stages. Moreover, the expression domains of PGC markers in *G. holbrooki* were either specific to teleost (*vasa* expression in developing PGCs), murine models (*dnd* spliced variants) or shared between the two taxa (germline and somatic expression of *piwi* and *nanos1*). Collectively, the results suggest that the reproductive developmental adaptations may reflect a transition from lecithotrophy to matrotrophy.

## Introduction

Primordial germ cells are the stem cells of life; they give rise to gametes, safeguarding the reproductive function of an individual and transfer its genetic material to ensuing generations. Two patterns of germ cell development are known in sexually reproducing animals; 1) Induction mode, where epigenetic reprogramming in a group of pluripotent cells induces PGC identity (Wang and Cao, 2016) and 2) Preformation mode, in which pre-packaged molecules in gametes, *i.e.,* germ plasm components, trigger PGC specification in early zygotes (Raz, 2002; Santos and Lehmann, 2004) which determine their germline fate and protect their identity against surrounding soma. Despite earlier thoughts, the germ plasm acquisition is not confined to primitive animals, but repeatedly shared in higher taxa (Ewen-Campen et al., 2010; Whittle and Extavour, 2017) particularly in those that exhibit lecithotrophy. With high species diversity, fish taxa have evolved both modes of PGC specification (Walvig, 1963; Extavour and Akam, 2003; Hansen and Pelegri, 2021). With majority of oviparous teleost including research models (Raz, 2002; Herpin et al., 2007) and commercial species (Nagasawa et al., 2013; Su et al., 2014) following the preformation mode, while most chondrichthyans (*i.e.,* cartilaginous fishes) following induction mode (Extavour and Akam, 2003) that also typically exhibit a degree of matrotrophy. However, little is known in poeciliids, which interestingly share aspects of both lecitho- and matrotrophy such as in the *G. holbrooki* (Mousavi and Patil, 2021).

Typically, PGCs undergo several developmental stages before transforming into functional germ cells. These begin with PGC specification regulated by parental derivates (Hong et al., 2016; Zhu et al., 2018) or epigenetic reprogramming (Yabuta et al., 2006; Saitou and Yamaji, 2012), followed by mobility acquisition to reach gonad anlage (Richardson and Lehmann, 2010; Paksa and Raz, 2015), and eventually attaining sexual identity, influenced by genetic/intrinsic (Devlin and Nagahama, 2002; Matsuda, 2005) or environmental/extrinsic (Baroiller et al., 2009) cues. Cumulative evidence suggests that each of these stages are governed by a group of molecules which recruit epigenetic machinery (Andersen et al., 2012), RNA interference apparatus (Giraldez et al., 2006) and/or signaling pathways (Boldajipour et al., 2008) safeguarding PGCs from somatic differentiation (Gross-Thebing et al., 2017), ectopic migration and apoptosis (Lai et al., 2012). Despite shared biochemical pathways, fundamental differences in the mode of PGC specification (*i.e.,* preformation vs induction) exists, as is obvious from spatio-temporal expression profiles of these molecules between taxa (Evans et al., 2014; Crother et al., 2016). For instance, during zebrafish (*Danio rerio*) embryogenesis, the maternally supplied *vasa* (Hartung et al., 2014) and *nanos1* (Koprunner et al., 2001) maintain the specificity and quantity of PGCs, respectively. Similarly, *dead end* (*dnd*), a regulator of the RNA interference machinery, is well conserved in teleost, whose maternal transcripts mark PGC sequestration and migration (Ketting, 2007) in models such as zebrafish (Weidinger et al., 2003), medaka (Hong et al., 2016) and Atlantic salmon (Nagasawa et al., 2013). However, in higher vertebrates with induction mode—*e.g.,* axolotl (Chatfield et al., 2014) and mouse (Saitou and Yamaji, 2010)—germ plasm does not form and the maternal deposit is erased in early stages of embryonic development (*i.e.,* cleavage). Instead, epigenetic machinery triggers PGC specification via bone-morphogenetic (BMP) signaling (Saitou et al., 2012; Seisenberger et al., 2012; Donoughe et al., 2014). Also, unlike medaka (Hong et al., 2016) and *Xenopus* (Taguchi et al., 2014), *Dnd* mutation (Sakurai et al., 1995; Youngren et al., 2005) and *Nanos1* ablation (Haraguchi et al., 2003) in mice do not affect PGC specification and fertility respectively. Therefore, despite the conserved regulatory pathways and set of molecules, PGC specification shows a shift in their developmental pattern in lecithotrophs with no maternal care (*e.g.,* zebrafish) and low maternal care, *e.g.,* medaka, (Morrison et al., 2017; Murata et al., 2020), to those distinct to matrotrophs (*e.g.,* mouse). Hence models such as poeciliids, *e.g.,* gambusia, with shared lecithotrophy (*i.e.,* where embryos receive nutrients only through the yolk) and matrotrophy (*i.e.,* where embryos receive nutrients from the mother) are likely to advance our current understanding as to how germline specification and maintenance evolve and function.

The ovo-viviparity of poeciliids is accompanied with interesting and complex alteration in their biology of reproduction including developing spermatozoa bundles (Grier et al., 1981) and an intromittent organ *i.e.,* gonopodia for deposition of sperm (Gasparini et al., 2011) in males, and matrotrophy, intrafollicular gestation (Pollux et al., 2014; Roberts et al., 2016) and superfetation (Norazmi-Lokman et al., 2016; Guzman-Barcenas and Uribe, 2019) in females. Moreover, the pseudo-placenta which evolved in poeciliids provides a maternal-fetal interface (Olivera-Tlahuel et al., 2018) sharing reproductive traits with placentalia. However, the consequences of this evolutionary convergence on germ cell formation and gonadogenesis is poorly understood.

In this regard, *G. holbrooki* presents as a particularly attractive poeciliid on count of its now wide global distribution (Norazmi-Lokman et al., 2021), short reproductive cycle (Norazmi-Lokman et al., 2016), well-documented embryology (Mousavi and Patil, 2021), close evolutionary relatedness to higher vertebrates (Furness et al., 2019; Furness et al., 2021) and notoriety as a pest fish (Ayres et al., 2012; Patil, 2012). A number of approaches to control its pest populations have been ineffective, particularly at large spatial scales. Hence, gaining insight into cellular and genetic pathways of germ cell development and gonadogenesis is expected to enhance our understanding of its reproductive biology to facilitate genetic control (Patil, 2012), whilst providing insights into comparative mechanisms of PGC development. This study for the first time examined PGC development across a range of embryonic stages of *G. holbrooki*, discerning shared features with other teleost and higher vertebrates, highlighting its utility to decipher comparative mechanisms of reproduction as well as direct application to control pest populations.

## Methods

### Wild Fish Collection and Housing

The fish were collected from the Tamar Island Wetland Reserve, Tasmania (41°23.1′S; 147°4.4′E) and reared in a dedicated small fish facility at Institute for Marine and Antarctic Studies (IMAS), Taroona, University of Tasmania. The rearing conditions are summarized in the Supplementary Table S1. The fish were fed with commercial pellets (TetraMin1 tropical granules, Germany) and freshly hatched Artemia nauplii (INVE Aquaculture, United States). The experimental ova and developing embryos were obtained from gravid females (Norazmi-Lokman et al., 2016) and staged (Mousavi and Patil, 2021) as previously described. All experimental procedures were approved by the University of Tasmania Animal Ethics Committee (Permit No. A12787).

### RNA Isolation and qRT-PCR

To evaluate the expression profile of key PGC markers during early development, total RNA from seven distinct developmental stages (Mousavi and Patil, 2021) including ovum (*i.e.,* vitellogenic oocytes stage V obtained from virgin females), cleavage (*i.e.,* mid-morula stage) blastula (*i.e.,* 1000-cell stage), gastrula (*i.e.,* the onset of embryonic shield elongation), early segmentation, late segmentation, and early pharyngula were isolated and used in quantitative PCR assay (n = 4-8 embryos/sex/developmental stage). To distinguish the spatial domains (*i.e.,* anterior and/or posterior of the developing embryos) of expression using end-point PCR, somite stage embryos (n = 5) were used. For this, most of the yolk was removed by puncture using Dumont #5 fine forceps (F.S.T, Canada) and the tissue was partitioned as head (anterior to otic vesicle) and trunk halves (Figure 2C) using a fine straight tipped dissecting knife (10,055-12, F.S.T, Canada), with each half processed separately for RNA isolation. The expression of the target genes in adult liver, kidney, spleen, skeleton muscle, heart, eyes, brain, testis and ovary (n = 4 sex/tissue) were also tested.

Individual embryos and adult tissues were rinsed in cold PBS, infiltrated with RNAlater (Sigma-Aldrich, Missouri, United States) and stored at -80°C until nucleic acid extraction. The RNA and DNA from individual embryos and 15–25 mg of tissues were isolated using AllPrep DNA/RNA Mini Kit (QIAGEN, United States). The residual genomic DNA in RNA extracts was removed using Ambion™ DNase (Thermo Scientific, United States) and RNA was purified by Monarch^**®**^ RNA Clean-up Kit (New England Biolabs). The quantity of isolated RNA was measured by Qubit^®^ four Fluorometer (ThermoFisher Scientific, Massachusetts, United States) and the RNA integrity was confirmed visually using agarose gel electrophoresis. The purified RNA was then reverse transcribed using MMLV Reverse Transcriptase (Takara, Kusatsu, Japan), with a final concentration of 6–230 and 250 ng/μl for embryos and tissues, respectively. The synthesised cDNA from adult tissues and somite embryos were later used in end-point PCR assays.

### cDNA Cloning and Full-Length Sequencing

To obtain full length cDNA of the genes from *G. holbrooki*, the predicted cDNA sequences from different poeciliid species were extracted from the NCBI database and multiple sequence alignments were applied using MUSCLE (Edgar, 2004). Subsequently, degenerate primers were designed based on highly conserved regions of the target cDNA homologues using Geneious Prime^®^ (version 2020.2.3). The primers were recruited to amplify target cDNA fragments through end-point PCR, the products were purified, cloned into pCR^®^2.1-TOPO^®^ vectors using TOPO™ TA Cloning Kit (Life Technologies Corporation, CA, United States) and sequenced. The resulting sequences were used to design *G. holbrooki* specific primers that were used in Rapid Amplification of cDNA Ends (RACE) PCR to obtain full-length cDNA using GeneRacer™ Kit (Life Technologies) according to the manufacturer’s protocol.

### Design of qPCR Primers and Data Normalisation

The qPCR assay primers (Supplementary Table S2) were designed using Geneious Prime^®^ (2020.2.3) with care to avoid self-dimer and secondary structures (Untergasser et al., 2012). Their efficiency was also tested at different dilutions for each gene, separately. The real time PCR mix (10 µl) comprised of 1X iTaq Universal SYBR Green Supermix (Bio-Rad, NSW, Australia), 5–10 ng cDNA Template, 0.4 µM of each primer, and adjusted to 10 µl using MilliQ water. Duplicate reactions were run for each cDNA sample using CFX96 Touch Real-Time PCR Detection System (Bio-Rad, NSW, Australia). Melting curve analysis, gel visualisation and sequencing of qPCR products were subsequently performed to check unwanted products and gDNA contamination.

Four housekeeping genes, namely *rps18*, *gapdh*, *pgk1* (Panina et al., 2018), and *β-actin* (Kwan and Patil, 2019) were tested for biological normalisation of qPCR data through geometric averaging of the candidate genes using geNorm algorithm (Vandesompele et al., 2002; Hellemans et al., 2007) in qbase + software (version 3.0, Biogazelle, Belgium). The *β-actin* with no sex-biased expression was selected to normalise qPCR data as the most stable housekeeping gene (M value 0.41). The relative transcription of target genes was calculated using the comparative threshold cycle (Cq) method with efficiency correction (Ruijter et al., 2009). Relative expression of genes of interest (∆Cq) was calculated against the selected reference gene and presented in plots. The expression fold changes presented were computed using the 2 ^(−∆∆Cq)^ method (Livak and Schmittgen, 2001).

### Statistical Analysis

The qPCR data was subject to analysis of variance (ANOVA) to identify significant differences between experimental groups. When applicable, Tukey HSD test used to compare the significance level of differences treatments. Normality of the data was tested with the Shapiro-Wilk test. The significance was set at *p* < 0.05.

### Whole Mount *in situ* Hybridization

The WM-ISH was used to investigate the spatial expression of target genes in several embryonic stages (*i.e.,* from late cleavage to early pharyngula). To ensure specificity, the sense and antisense RNA probes were generated from a less conserved domains such as untranslated regions (Thisse and Thisse, 2008). The respective cDNAs were first inserted into pCR^®^2.1-TOPO vector. DIG-labelled RNA probes were produced by *in vitro* transcription using T7/T3 RNA polymerase (NEB) and DIG RNA labelling mix (Roche, Mannheim, Germany). Any traces of cDNA in the probe were eliminated using Ambion^**TM**^ DNase (Thermo Scientific, United States), purified by ethanol precipitation and stored with RNase inhibitor, RNasein^®^ Plus (Promega, United States) at -20°C.

The WM-ISH followed those described for small fish embryos (Thisse and Thisse, 2008) with modifications. Briefly, the dissected clutches of developing embryos were individually detached from placenta, rinsed with cold PBS, and fixed using 4% paraformaldehyde (Emgrid) overnight at 4°C. The fixed embryos were washed in PBS containing 0.1% Tween 20 (PBT), progressively dehydrated with PBT-methanol, and stored in 100% methanol until use. The embryos at early and mid-pharyngula stages were depigmented before dehydration using 3% H_2_O_2_ and 1% KOH. On the day of hybridization, the embryos were sequentially rehydrated in four stages with progressively increasing concentrations of methanol-PBT, manually dechorionated, permeabilized with 10–25 μg/ml proteinase K (Bioline) and postfixed with 4% paraformaldehyde for 30 min. The embryos were prehybridized at 68°C for 3 h in hybridization buffer (50% formamide, 5X SSC, 0.01% Tween 20, Torula Yeast tRNA, 50 μg/ml heparin) and hybridized in fresh buffer containing antisense RNA probes (100–250 ng/ml) at 67–69°C for 16–24 h, as required for target gene and developmental stage. This was followed by stringency washes in PBST, with progressively lower salt concentrations, to remove any non-specifically bound probes. For immuno-labelling, non-specific binding was first prevented using blocking solution (5% blocking reagent (Roche) in maleic acid buffer containing 0.1% Tween 20). The embryos were then treated with 1:3,000–1:5,000 anti-DIG alkaline phosphatase (AP) antibody (Roche) at 4°C for 16 h. The antibody-labelled embryos were washed with PBT 8 times, 30 min each, at room temperature, under gentle agitation. For staining, the embryos were first treated with staining buffer (100 mM Tris HCl pH 9.5, 50 mM MgCl2, 100 mM NaCl, 0.1% Tween 20) three times by replacing with fresh buffer every 10 min and then incubated in BM-purple stain (Roche) at room temperature and protected from light. The optimum staining time was adjusted according to developmental stages and target genes ranging from 5 to 35 h. Following staining, the embryos were washed in PBT until all excess stain was removed and postfixed with 4% paraformaldehyde overnight at 4°C. The fixed embryos were washed and stored in PBS for imaging using MZ16FA stereomicroscope (Leica Microsystems, Germany).

### Genetic Sexing of Embryos

The isolated DNA (co-extracted with RNA using AllPrep DNA/RNA Mini Kit) were used for genetic testing as previously described for this species (Kwan and Patil, 2019; Patil et al., 2020). Briefly, PCR mix (10 µl) comprised of 1X MyTaq™ HS Red mix (Meridian Life Science, OH, United States), 0.4 µM of each primer and 50 ng of genomic DNA template. Thermal cycling (T100™ Thermal Cycler, Bio-Rad Laboratories, NSW, Australia) consisted of 95°C for 1 min, followed by 30 cycles of 95°C for 5 s, 60°C for 5 s, and 72°C for 20 s. Female and male specific amplicons were visualised using gel electrophoresis.

## Results

### The Pattern of PGC Migration in *G. holbrooki*

**FIGURE 1:**
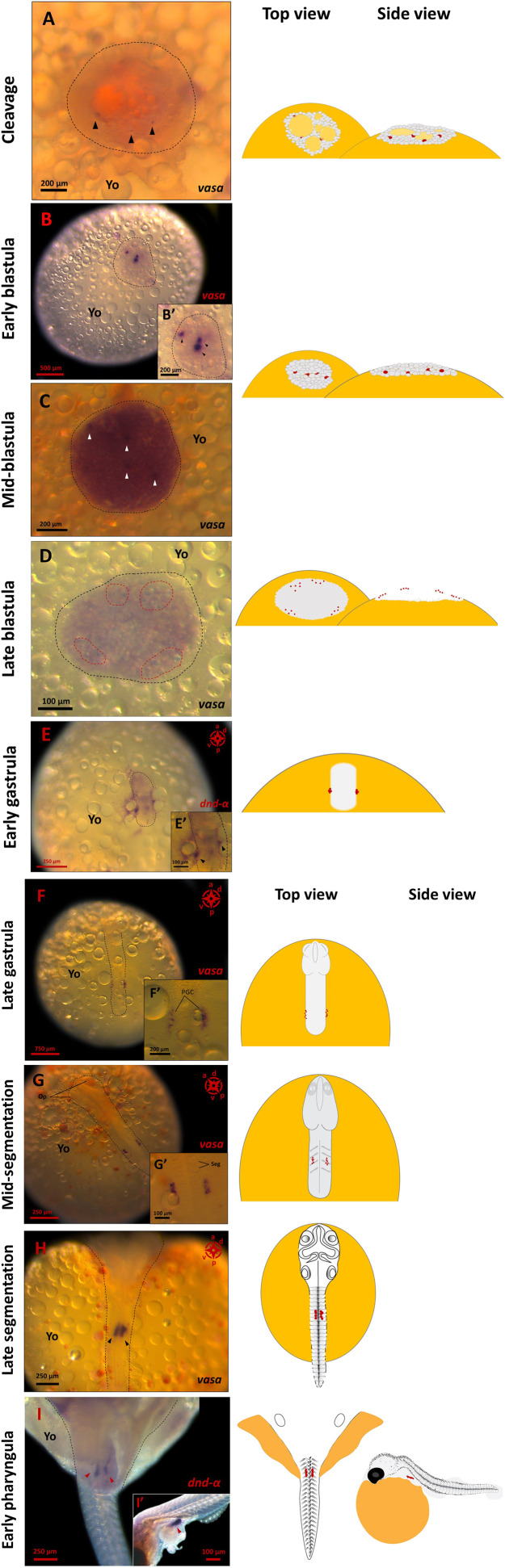
PGC formation and migration in *G. holbrooki*. WISH panels showing the actual and schematic locations of PGC during *G. holbrooki* embryogenesis. The PGC markers, *vasa* or *dnd-α* signals at nine developmental stages are presented. The *vasa*-positive cells are first detectable (**(A)**, black arrowhead) emerging as a few tiny spots distributed throughout the cell mass (n = 3). At early blastula **(B)**, the *vasa* signal was intensified in a few distinctive regions (black arrowhead in close-up B′) with asymmetric distribution (n = 4). Later, four distinctive spots (**(C)**, white arrowhead) are detectable buried underneath the superficial layer of cells (n = 3). At late blastula **(D)**, the *vasa*-positive signals increase and appear peripherally as small clusters (n = 4). At early gastrula **(E)**, the PGCs coalesce as two compact clusters of cells (black arrowhead in the close-up E′) located at the margin of the dome (n = 4). At late gastrula **(F)**, the PGCs were located at the posterior half of the embryo (n = 3). During segmentation stages (**(G)**, n = 5 and **(H)**, n = 4), the PGC clusters migrate convergently and anteriorly, and at early pharyngula **(I)**, they eventually home at genital ridge (red arrowheads) and form a bi-lobular presumptive gonad (n = 7). The black dashed lines indicate the area of the cleavage plane and blastodisc **(A,B)**, and embryonic axis **(E,F,G,H,I)** respectively. The red dashed lines **(D)** show the signals clustered in four groups. The red dots in schematics represent relative position of the PGC marker signals. Op, optic bud; Yo, yolk; Seg, segments.

The *vasa* mRNA signals were first detectable in late cleavage stages, emerging as a few tiny spots distributed throughout the blastomere (Figure 1A). At this stage, the signals were partially obscured by egg yolk (*i.e.,* meroblastic cleavage) and buried deep in the cells mass, partially masked by glare of the large oil-droplets beneath. At early blastula (1000-cell stage), concurrent with increased blastoderm area (400–450 µm) and asynchronous cell divisions, the *vasa* signal was intense at three distinct spots that were asymmetrically distributed with one larger and darker than the other two (Figure 1B). At mid-blastula, four distinctive spots were detectable buried underneath the layer of cells at almost the same geometrical plane (Figure 1C). At late blastula, the *vasa*-positive signals had numerically increased and arranged peripherally to the blastodisc in four clusters (Figure 1D).

At the onset of the gastrula stage (*i.e.,* corresponding to 50%-epiboly in zebrafish and egg cylinder in mice), the blastoderm had begun to expand within a crater-like depression over the surface of the yolk at the animal pole. Here, the *dnd-α* signals had coalesced and appeared as two compact clusters of cells located at the margin of the dome (Figure 1E) where both the dome and the yolk boundaries were difficult to distinguish and so were the anterior-posterior axis (Figure 1E). For the first time, from mid to late gastrula, the anterior-posterior axis of the embryo was distinguishable and the optic primordia began to form as the body-axis lengthened and thickened. Here, the *vasa*-positive cells appeared pellucid and were located at the posterior half of the embryo. At this stage, the PGCs were clustered at the junction of developing embryo and the yolk, close to posterior of the trunk, however, the individual germ cell precursors were distinguishable (Figure 1F).

At the onset of somitogenesis, concurrent with considerable expansion of the head, rudimentary brain formation and first visible somites, the two clusters of PGCs were still visible, one on either side of the body axis, forming narrow queue of cells (Figures 1G, 4D). Based on the spatial distribution of PGCs two directions of motility were discernible, with the two clusters beginning to 1) converge inwards, *i.e.,* PGC clusters moved towards each other and 2) migrating anteriorly.

As evident from migrating signals at early to mid-somitogenesis, coinciding with significant body elongation, PGCs exhibited a relatively enhanced mobilisation compared to late gastrula stage. Specifically, at 12–14 somite stage, where solid optic capsule and otic vesicles were also visible, the two PGC clusters were clearly observed extending from the sixth to eighth somite (Figure 1G). The PGC clusters maintained their integrity (*i.e.,* cells moving in a close vicinity to each other), however, the narrow queue of cells progressively clustered tightly (thickened and shortened cluster of cells), compared to preceding developmental stage. At late segmentation, PGC clusters were observed on either side of the body axis, directly underneath the somites and the spinal cord, in closest vicinity to each other (Figure 1H). At early pharyngula, the PGCs had migrated and coalesced at the genital ridge and remained in two distinctive clusters (Figure 3E).

### Spatio-Temporal Expression of PGC Markers in *G. holbrooki*

**FIGURE 2:**
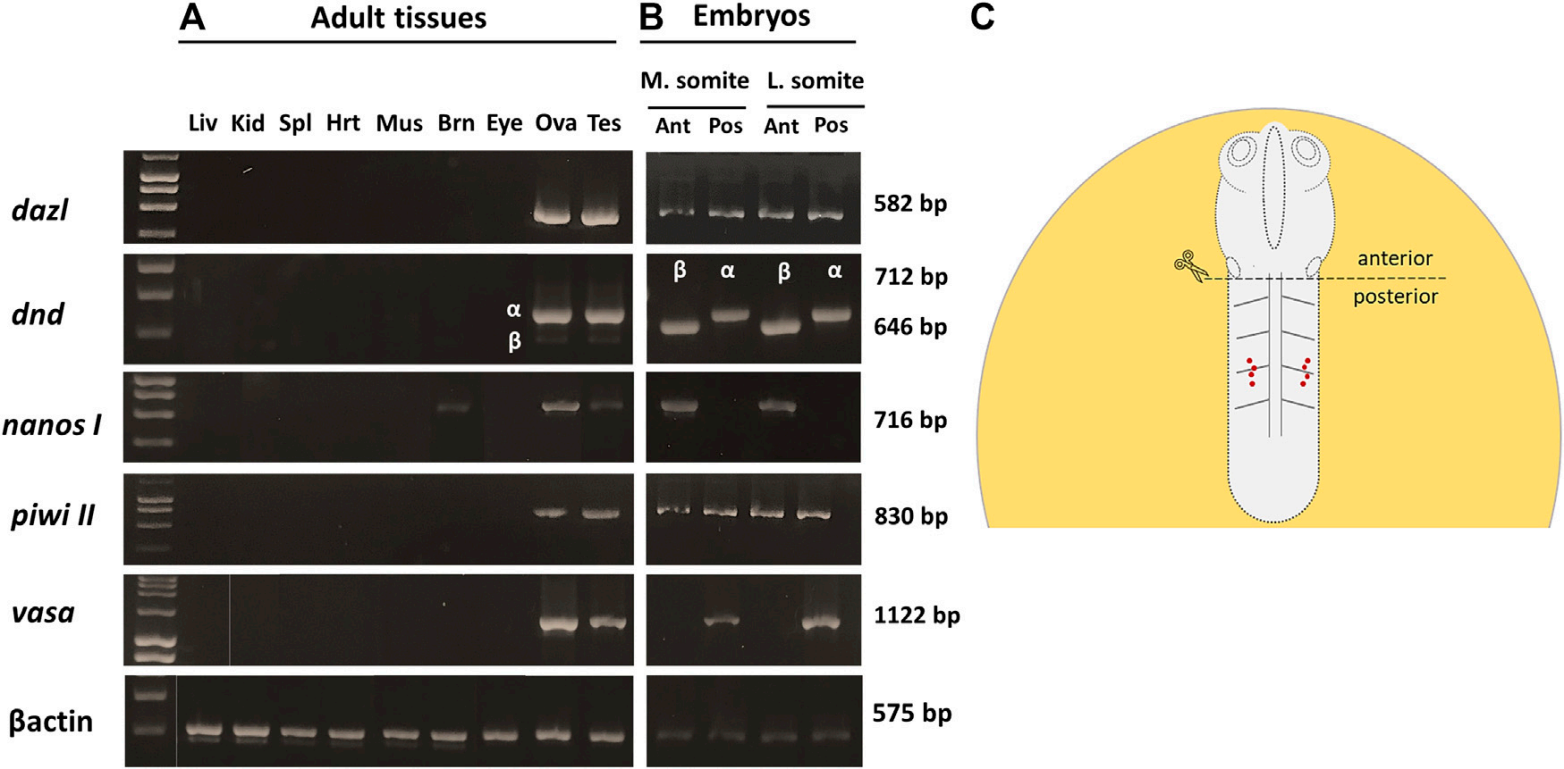
The spatio-temporal expression of PGC markers in pre- and postnatal *G. holbrooki*. Photomicrograph, showing the expression of PGC marker genes in adult tissues, 75 days post parturition **(A)** and embryos at mid-somite stage **(B)**. Gene names and the amplicon size, in base pairs (bp) are indicated on the right and left of the panel respectively. *βactin* was used as endogenous reference. The schematic **(C)** shows anterior and posterior partitioning of the somite stages embryos for RNA extraction and end-point PCR assay. The red dots **(C)** show the location of migrating PGCs in mid-segmentation embryos. Liv, liver; Kid, kidney, Spl, spleen; Hrt, heart; Mus, skeletal muscle; Brn, brain; Eye, eyes; Ova, ovary; Tes, testis; M. somite, mid-somitogenesis; L. somite, late somitogenesis; Ant, anterior; Pos, posterior.

**FIGURE 3:**
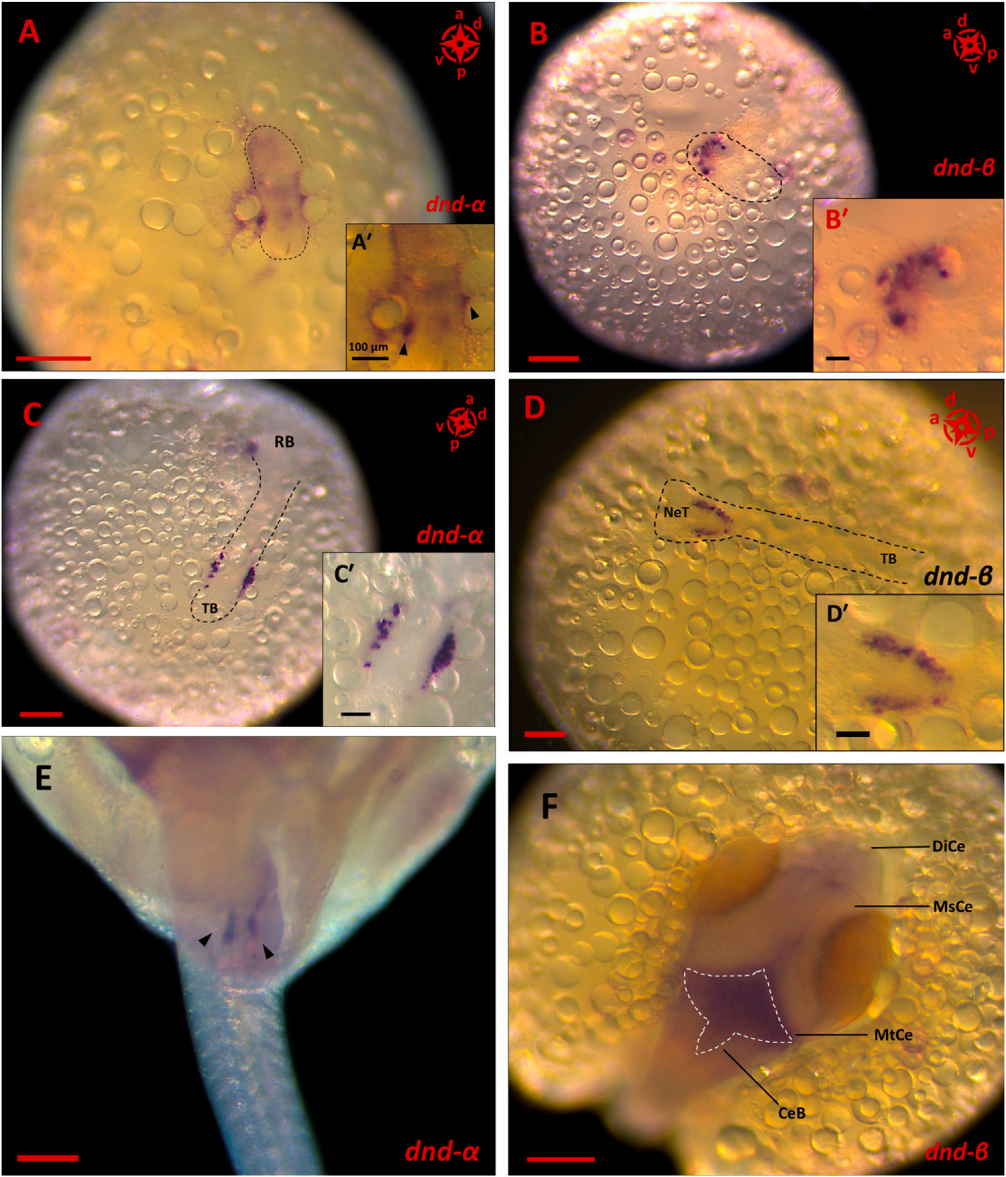
The expression pattern of *dnd* spliced variants in embryonic stages of *G. holbrooki*. WISH panels showing spatial expression of *dnd* spliced variants at three developmental stages of *G. holbrooki*. *dnd-α* is expressed in the posterior region of early gastrula (**(A)**, and close up inset, n = 4), with condensed signals at two spots (black arrowheads in A′) in the peripheral embryonic shield. At early segmentation (n = 4), two clusters of *dnd-α* signals (corresponding to the location of PGCs) later appeared close to the tail bud (TB) (**(C)** and close up inset, n = 4). At early pharyngula, *dnd-α* transcription marked the homed germ cells (black arrowheads) located as two distinctive lobes at the genital ridge (**(E)**, n = 7). While the *dnd-β* was first detected at the anterior region of the elongating body (**(B)** and close up inset, n = 4). The *dnd-β* signal emerged as a crescent line at the putative head of gastrula (n = 3) but expanded in both sides of the neural tube (NeT) at early segmentation (**(D)** and close up inset, n = 6) and eventually marked the metencephalon (MtCe) and cerebellum (CeB) of early pharyngula (**(F)**, n = 8) with no expression in Mesencephalon (MsCe) and diencephalon (DiCe). The compass indicates the orientation of body axis where applicable (a, anterior; p, posterior; d, dorsal and; v, ventral). The black dashed lines indicate the location of embryonic shield **(A,B)** or body **(C,D)**. The white dashed line displays the expression area of *dnd-β* in hindbrain. The scales represent 100 and 250 µm in **(A′–D′)** and **(A–F)**, respectively.

**FIGURE 4:**
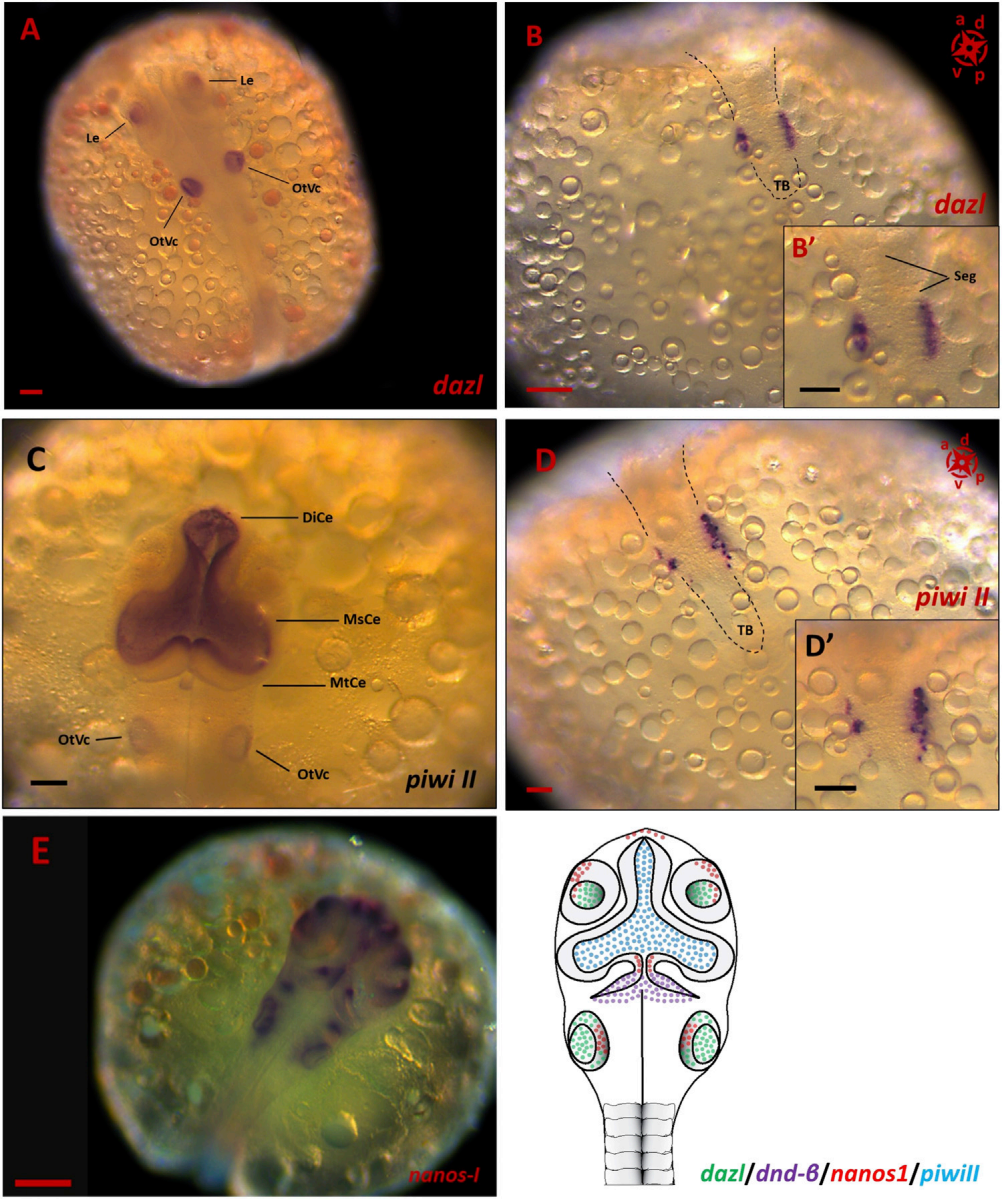
The spatial expression of PGC markers in embryonic stages of *G. holbrooki*. WISH panels showing spatial expression of *dazl* **(A,B)**, *piwi II* **(C,D)** and *nanos1* **(E)** during somitogenesis in *G. holbrooki*. The *dazl* expression (n = 5) was detected in otic vesicle (OtVc) and optic lens (Le), anteriorly **(A)** and PGC clusters posteriorly **(B)** and close up inset), in a closer proximity to tailbud (TB), right in both sides of segments (Seg). In slight contrast, the anterior expression of *piwi II* (n = 7) was strongly detected in the region of developing brain predominantly in diencephalon (DiCe) and mesencephalon (MsCe) with a very weak expression in otic vesicles. Whereas the posterior expression of *piwi II* was restricted to migrating PGCs (**(D)** and close up inset). The *nanos1* expression was exclusively detected in developing brain and otic vesicle of mid-somitogenesis embryos (n = 6). The compass indicates the orientation of developing embryos where applicable. The schematic **(F)** represents the simultaneous expression domains of PGC markers in CNS during somitogenesis. The black dashed lines indicate the location of embryonic body. The scale bars represent 100 µm.

Of all the PGC markers, only *dnd* had the two spliced variants; the longer variant, *dnd-α*, contained five exons with 1,122 bp CDS, while the shorter *dnd-β* (1,056 bp CDS) contained six exons. Based on end-point PCR, all the candidate PGC markers were expressed in adult ovary and testis, with additional expression of *nanos1* in brain (Figure 2A). As for the embryonic expression, two of which were detected in the posterior (*vasa* and *dnd-α*) and anterior (*nanos1* and *dnd-β*) halves of somitogenesis embryos, while *piwiII* and *dazl* were detected in both consistent with WMISH observation (Figures 2B,C). More specifically, *vasa* (Figure 1), *dnd-α* (Figures 3A,C,D), *dazl* (Figure 4B) and *piwiII* (Figure 4D) transcripts expressed in the posterior half overlapped with the location of nascent PGCs. While the anterior expression of *dazl* was found as strong signal in the otic vesicles with a weak signal in the eyes (Figure 4A). The anterior expression of *piwiII* corresponded to large part of the developing brain but restricted to mesencephalon and diencephalon (Figure 4C). The *dnd-β* was first detected at the anterior region at early gastrula (Figure 3B), then expanded on both sides of the developing brain at early segmentation (Figure 3D) and cerebellum during early pharyngula (Figure 3F).

**FIGURE 5:**
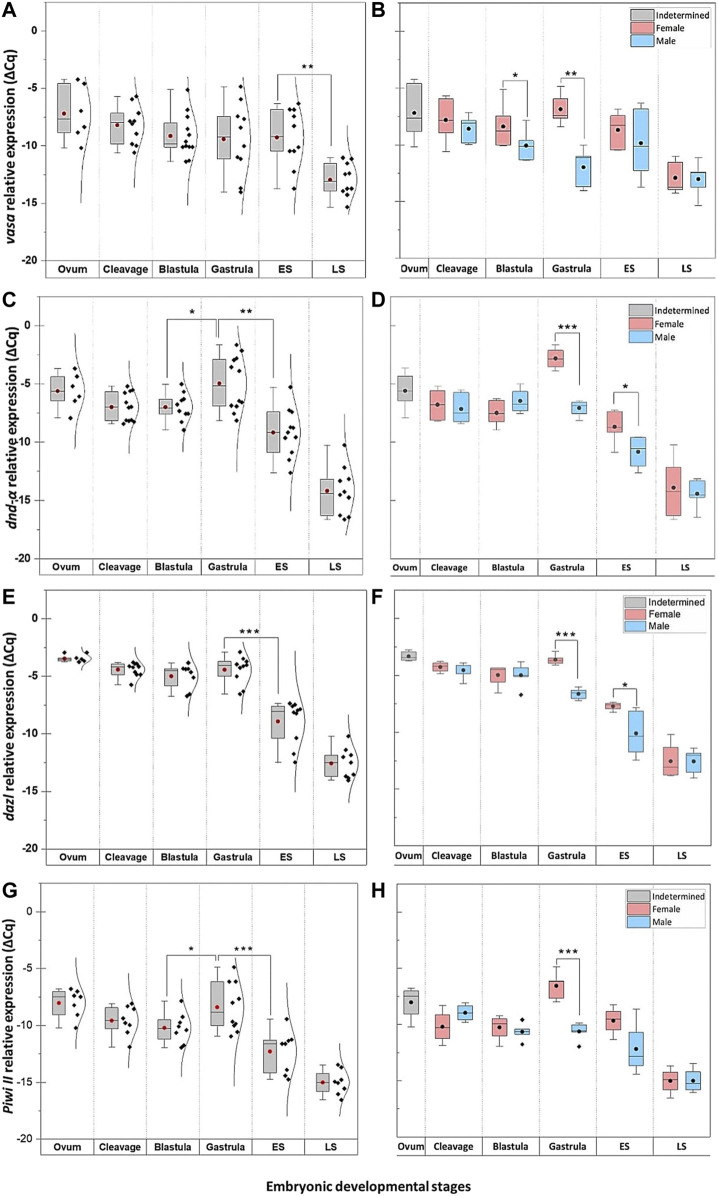
The temporal expression of PGC markers at select embryonic stages of *G. holbrooki*. The pooled (left panel) and sex-segregated (right panel) quantitative expression of *vasa* **(A–B)**, *dnd-α* **(C–D)**), *dazl* **(E–F)**, and *piwiII* **(G–H)** in six embryonic developmental stages of *G. holbrooki*. ES, early somitogenesis; LS, late somitogenesis. The dots and the horizontal line within the bars indicate mean and median of the data presented within the groups. The normal distribution curves are given in the pooled expression plots. The asterisks show the level of significance between groups; Tuckey’s HSD, * = 0.05, ** = 0.01, *** = 0.001.

**FIGURE 6:**
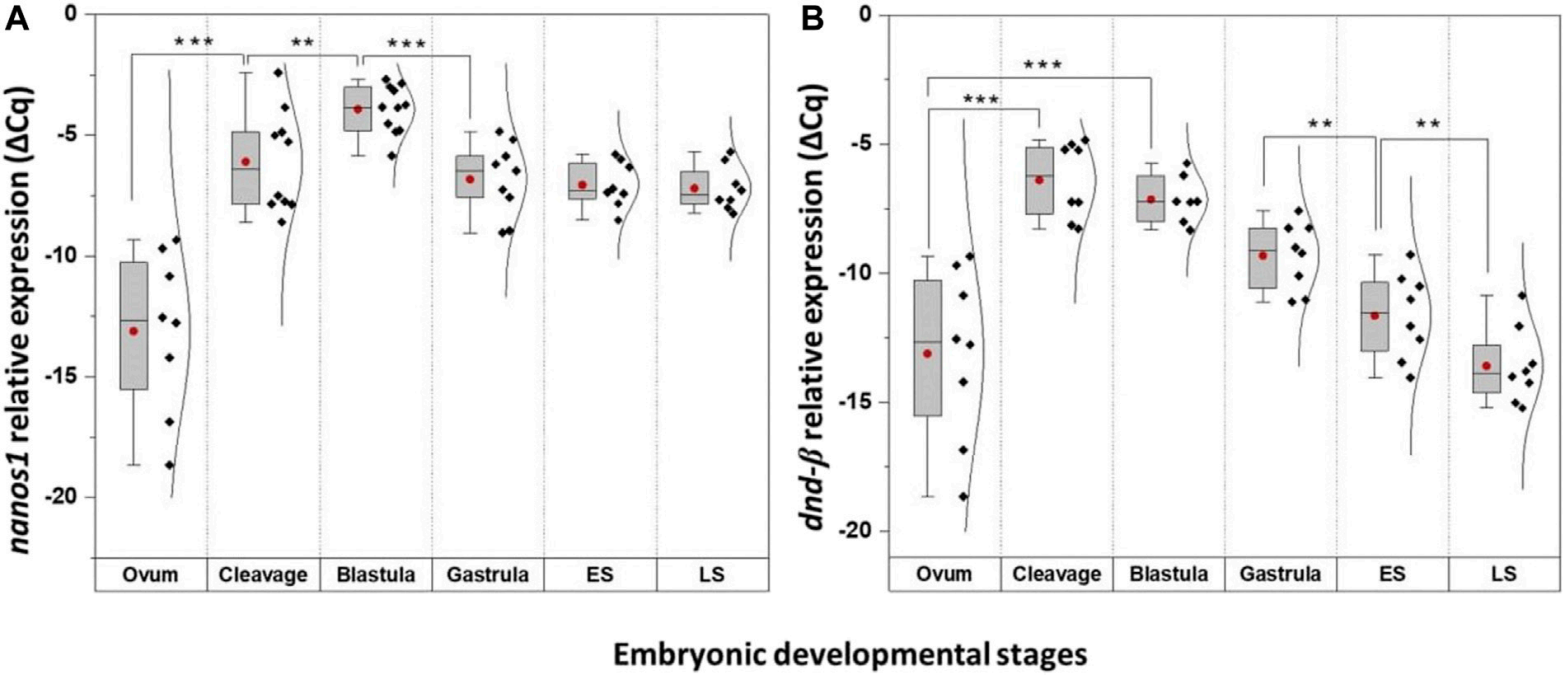
The temporal expression of *nanos1* and *dnd-β* at select embryonic stages of *G. holbrooki*. The panel representing quantitative expression of *nanos1* **(A)** and *dnd-β* **(B)** in six embryonic developmental stages of *G. holbrooki*. ES, early somitogenesis; LS, late somitogenesis. The dots and the line within the bars indicate mean and median of the data presented within the groups. The normal distribution curves are given in the global expression plots. The asterisks show the level of significance between the marked groups; Tuckey’s HSD, * = 0.05, ** = 0.01, *** = 0.001.

Quantitatively, four of these PGC markers (*dnd-α*, *dazl*, *piwi II*, and *vasa*) were detected in unfertilised egg (Figure 5), with *nanos1* and *dnd-β* barely detectable or absent (Figure 6 and Table 1). The maternally expressed genes showed a comparable trend in cleavage and blastula (Table 1). However, the maternally silent genes were strongly upregulated (*p* < 0.05, Table 1) at early embryogenesis (*i.e.,* cleavage). Comparing the quantitative expression of maternally inherited genes between stages (Table 1), a significantly transient surge was observed at gastrula stage (*p* < 0.05) with a female-biased trend (*p* < 0.05).

**TABLE 1:** Spatio-temporal expression of PGC-specific markers at select embryonic stages of *G. holbrooki*.

Genes	Spatio-temporal Expression		Ovum	Cleavage	Blastula	Gastrula	Early somitogenesis	Late somitogenesis	Figures
*Vasa*	Qualitative		—	- Tiny spots in blastomere (1A)	- Four clusters at the periphery of the blastodisc (1D)	- Two clusters at the margin of dome (1E and 1F)	- Posteriorly, at migrating PGC clusters (1G)	- PGR (1I)	Figures 1, 2B
	Quantitative	Between stages	−1.0 ± 1.0−0.94 ± 0.75−0.28 ± 1.06−3.69 ± 0.91**+0.14 ± 1.26						Figure 5A
		Between sex	—	0.78 ± 0.08 F	2.68 ± 1.02 F *	5.12 ± 1.01 F **	1.53 ± 2.03 F	0.11 ± 0.97 F	Figure 5B
*dnd-α*	Qualitative		—	- Tiny spots in blastomere	- Four clusters at the periphery of the blastodisc	- Two clusters at the margin of dome at vasa-positive cells (3A)	- Two PGC clusters close to the tail bud (3C)	- PGR (3E)	Figures 1, 2B, 3
	Quantitative	Between stages	−1.38 ± 0.66−0.01 ± 0.52+2.4 ± 0.81 *−4.21 ± 0.92 ***−5.2 ± 0.89 ***						Figure 5C
		Between sex	—	0.36 ± 0.73 F	1.70 ± 0.70 M	4.25 ± 0.42 F ***	2.15 ± 0.89 F *	0.52 ± 0.38 F	Figure 5D
*dnd-β*	Qualitative		—	—	—	- Anterior region of the elongating body (3B)	- Both sides of the neural tube (3D)	- Metencephalon and cerebellum (3F)	Figures 2B, 3
	Quantitative	Between stages	+6.71 ± 1.27 ***−0.74 ± 0.61−2.17 ± 0.61 *−2.32 ± 0.76 **−1.95 ± 0.78 *						Figure 6A
*Dazl*	Qualitative		—	- Tiny spots in blastomere	- Four clusters at the periphery of the blastodisc	- Two clusters at the margin of dome at vasa-positive cells	- Anteriorly, at optic lens and otic vesicles (4A). - Posteriorly, at PGC clusters (4B)	- Otic vesicles - Optic lens - PGR	Figures 2B, 4
	Quantitative	Between stages	−0.96 ± 0.28−0.57 ± 0.39+0.56 ± 0.53−4.49 ± 0.70 ***−3.64 ± 0.75 **						Figure 5E
		Between sex	—	0.28 ± 0.38 F	0.02 ± 0.72 M	3.01 ± 0.31 F ***	2.39 ± 0.94 F *	0.01 ± 0.93 M	Figure 5F
*piwiII*	Qualitative		—	—	- Four clusters at the periphery of the blastodisc	—	- Anteriorly, at diencephalon and mesencephalon (4C). - Posteriorly, at PGC clusters (4D)	- diencephalon and mesencephalon, - PGR	Figures 2B, 4
	Quantitative	Between stages	−1.53 ± 0.62−0.63 ± 0.65+1.85 ± 0.86 *−3.9 ± 0.95 F ***−2.71 ± 0.75 **						Figure 5G
		Between sex	---	1.24 ± 0.83 M	0.40 ± 0.65 F	4.09 ± 0.67 F ***	2.5 ± 1.36 F	0.01 ± 0.80 F	Figure 5H
*nanos1*	Qualitative		—	—	—	- A single cluster at the anterior half	- Neural tube (4E) - Otic vesicles (4E)		Figures 2B, 4
		Quantitative	Between stages	+7.02 ± 1.27 ***+2.16 ± 0.7 **−2.89 ± 0.55 ***−0.23 ± 0.61−0.15 ± 0.45						Figure 6B

Level of significance between groups (∆∆Cq, Tuckey’s HSD): * = 0.05, ** = 0.01, *** = 0.001.

The ∆∆Cq between sexes were not significant for *nanos1* and *dnd-β* at any stage.

## Discussion

While a previous study (Pala, 1970) investigated PGCs in *G. holbrooki*, this was limited to late developmental stages and gross cell morphology. In contrast, a detailed PGC developmental dynamics presented here captures shared evolutionary features of both lecitho- and matrotrophic vertebrates.

### Evidence for Maternal Inheritance of PGC Specification

The relatively high expression of PGC markers tested (*dazl*, *dnd-α*, *piwi II*, and *vasa* but not *nanos1*) in unfertilised eggs of *G. holbrooki* implies their maternal inheritance and suggests PGC specification is determined by preformation mode. This pattern is similar to those observed in oviparous species such as zebrafish (Kaufman and Marlow, 2016) and medaka (Herpin et al., 2007) as well as those of *Drosophila* (Rongo and Lehmann, 1996), and *Xenopus* (Chan et al., 2007)*.* However, in mice, mechanical ablation of vegetal or animal pole of the fertilised egg did not arrest the development of viable and fertile offspring (Zernicka-Goetz, 1998). This was later confirmed by the evidence that nuage was not detectable in unfertilised and fertilised murine eggs (Toyooka et al., 2000) suggesting PGC specification is governed by zygotic determinants in mice.

In teleosts, the maternal factors such as *tdrd6* safeguards proper germ plasm segregation (Roovers et al., 2018), while, *dnd* (Hong et al., 2016; Zhu et al., 2018) and *dazl* (Li et al., 2016) are necessary for maintaining PGC identity, with *nanos1* (Koprunner et al., 2001), and *vasa* (Braat et al., 2001; Li et al., 2009) providing signals for their proliferation and correct migration. The maternal accumulation of these markers in ova supports early germline sequestration in *G. holbrooki* with comparable functions reported for other fish models.

### Relatively Early Onset of Zygotic Activation Mimics Those of Mouse

The presence of *nanos1* and *dnd-β* transcripts in post-fertilisation embryos, but not in ova suggests its zygotic origin. Their first appearance at mid-cleavage is a preliminary evidence of zygotic genome activation (ZGA) onset which is earlier compared to what occurs at mid-blastula in zebrafish (Jukam et al., 2017). By inference, this appears a relatively early model of global zygotic activation among teleosts. The timing of MZT is not conserved across species nor follows a pattern of evolutionary hierarchy. For example, the earliest ZGA has been reported in sea urchin (Tu et al., 2014) and mice (Abe et al., 2015) at first cell division, while in zebrafish (Pelegri, 2003; Lindeman and Pelegri, 2010; Aanes et al., 2011) and *Drosophila* (Atallah and Lott, 2018), its activation is postponed until the 10th and eighth cycles, respectively. Typically, the onset of global ZGA are evaluated by an array of cellular events. For example, cell cycle intervals lengthen, as zygotic transcription interrupts DNA replication (Rothe et al., 1992), coinciding with the initiation of ZGA. However, in species with relatively early ZGA (e.g., mice and *G. holbrooki*), demarcation of ZGA with longer cell cycle intervals are less likely to be useful. Instead, more refined indicators such as the appearance of maternal transcript repressors (Ruzov et al., 2004), increased chromatin accessibility (Liu et al., 2018) and dilution of histone concentration (Joseph et al., 2017) may provide greater precision.

### Epigenetic Trigger May Explain PGC Markers Surge

Activation of PGC markers (*dazl*, *piwi II,* and *dnd-α*) at gastrula in *G. holbrooki* is similar to those in mice (Smith and Meissner, 2013) and zebrafish (Potok et al., 2013), known to be initiated via epigenetic mechanisms. Specifically, the second major wave of epigenetic reprogramming in mice triggers around E6.5 (at the onset of gastrula) where PGCs are liberated from methylation (Gao and Das, 2014). This is simultaneous with PGC extrusion from the epiblast, *i.e.,* their specification from soma, *en route* to the genital ridge (Lee et al., 2014). In zebrafish, the pattern of DNA methylome is not fully comparable with those of mice (Potok et al., 2013), however, germ cell specific markers (e.g., *piwi*, *dazl,* and *vasa*) undergo hypomethylation post-ZGA during the sphere stage (Seisenberger et al., 2012; Potok et al., 2013; Hill et al., 2018). The timing of these two events is concurrent with the surge in expression of *dazl* and *piwi II* at gastrula in *G. holbrooki*, a likely consequence of demethylation wave in the germline. However, this needs verification.

### Expression Profile of Germline Markers Imply Sex-Dimorphic Proliferation of PGCs

A transient female-biased expression of PGC markers in *G. holbrooki* is similar to those of zebrafish (Ye et al., 2019) where preferential PGC proliferation leads to female differentiation. In zebrafish*,* the timing of PGC proliferation is known to occur as early as gastrula (Feng et al., 2020) which begins between sphere and 50% epiboly (Wang et al., 2016). This parallels the evanescent upregulation of the markers in *G. holbrooki*, which likely corresponds to female-biased PGC proliferation.

### PGC Migration Pattern Shares Features of Both Egg Laying Teleosts and Mouse

**FIGURE 7:**
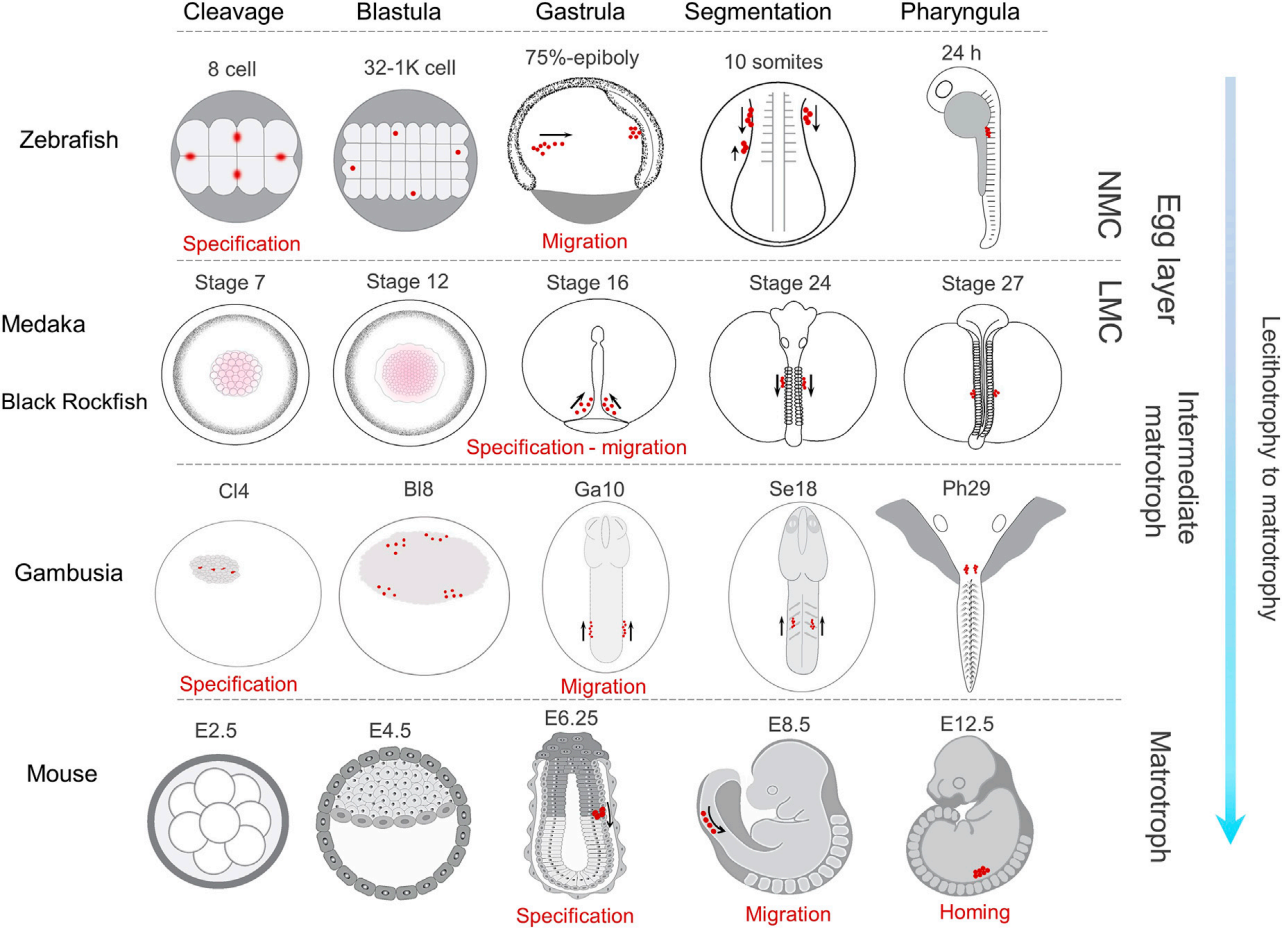
Comparative PGC development in representative lecithotrophic and matrotrophic species with increasing level of maternal care. In zebrafish (Raz, 2003; Eno and Pelegri, 2013) and *G. holbrooki*, PGC markers are compartmentalised in four distinct cells (red dots) by cleavage (8-cell and Cl4, respectively). They later form four clusters in Blastula (32-1K Cell and Bl8 stages, respectively). In gambusia, two each of the four clusters merge at gastrula (Ga10) and appear as a cluster each on either side of the body axis, while in zebrafish this rearrangement is postponed to 10 somites. In medaka (Herpin et al., 2007) and black rockfish (Zhou et al., 2020), the Gp markers are dispersed throughout the cytoplasm of blastomeres (stages 7 and 12) until gastrula where the *nanos*-positive cells are evident at stage 16, forming two clusters in either side of the body. In mice, PGC specification is first evident in egg cylinder stage (E6.25) and the PGCs remain in a single cluster until their colonisation (Saitou and Yamaji, 2010). The onset of PGC migration in all the four species occurs at gastrula, albeit slightly delayed in mice. However, the pattern of their migration is somewhat dissimilar due to developmental constraints specific to the respective species. In all the four teleosts, during segmentation, PGC clusters coalesce in either side of the body axis and migrate towards PGR. In contrast only one cluster forms in mice, an arrangement persisting until PGC colonisation (at E12.5). The direction of PGC migration at segmentation is predominantly posterior in both zebrafish and medaka, while this is anterior in *G. holbrooki* and mice. NMC, no maternal care; LMC, low maternal care.

Consistent with the gene structure and content, the *vasa* and *dnd-α* expression in gambusia was restricted to the domains corresponding to PGCs. This was further supported by the restricted localisation of *vasa* transcript at the genital ridge of parturating embryos, where the newly formed gonad emerges. Although the molecular components and the machineries involved in PGC mobilisation are conserved among teleosts, the pattern of their migration varies between species (Saito et al., 2006). Typically, the early segregation of PGCs in teleosts relies on the localisation pattern of germ plasm, as the maternally inherited germ cell determinants (Bontems et al., 2009; Roovers et al., 2018). However, two types of germ plasm arrangements have so far been described in early embryonic development of teleosts where (Figure 7); 1) germ plasm components aggregate compactly in cleavage furrows (Yabe et al., 2009) until the 16-cell stage, subsequently segregating into proliferating cells eventuating in four clusters of germ plasm-positive cells at the dome stage as in zebrafish (Raz, 2002), Atlantic cod, *Gadus morhua* (Presslauer et al., 2012), and olive flounder, *Paralichthys olivaceous* (Li et al., 2015) while in others, 2) the germ plasm signal is dissipated throughout the cell mass in early cell cycles with distinct germ plasm-positive cells first forming at the mid-gastrula stage, such as in medaka (Herpin et al., 2007). Although, the early cleavage stages could not be captured, the occurrence of four loosely aggregated clusters of *vasa*-positive cells at the periphery of blastodisc in *G. holbrooki* follows the former pattern (Figure 7).

The position of PGCs determines their migration start point which is defined in relation to cell collectivity and body patterning during blastula and gastrula stages, respectively (Raz, 2002). In zebrafish (Kimmel et al., 1995), medaka (Iwamatsu, 2004), and viviparous black rockfish (Zhou et al., 2020), prominent embryonic features (*e.g.,* sphere, yolk cell doming, multiple stages of epiboly) during blastula stage pose broad somatic environment for cell motility. While in poeciliids including *G. holbrooki* (Tavolga and Rugh, 1947; Tavolga, 1949; Mousavi and Patil, 2021), the blastodisc is extremely thin and flat and the growing cells sink inwards into the emerging yolk cavity. Therefore, cell patterning occurs in a relatively limited environment independent of yolk as a scaffold (dissimilar to zebrafish and medaka and similar to mice) where blastodisc does not grow over yolk cell to form dome. Indeed, the embryonic shield in *G. holbrooki* is relatively compact and at early gastrula swiftly begins to elongate and form embryonic body axis in the growing but restricted cavity on the yolk surface (Mousavi and Patil, 2021). The dissimilarity in early embryonic body patterning (during blastula and gastrula) may affect the expression domain of chemokine signalling (*e.g.,* Cxcl12a-Cxcr4 interaction) as the key regulator of PGC migration in vertebrates. The formation of two clusters at early gastrula in gambusia, is comparable to the initial patterning of nascent germ cells in medaka (Saito et al., 2006; Kurokawa et al., 2007) where the PGCs are first found dispersed on both sides of the dorsal axis (Herpin et al., 2007).

During somitogenesis, PGC clusters in teleosts show two spatial patterns while migrating towards PGR. In one group including zebrafish (Weidinger et al., 1999), loach (Saito et al., 2006), and medaka (Herpin et al., 2007), the PGCs form two clusters positioned on either side, of the trunk and moving separately towards the genital ridge. In the second group including herring and floating goby (Saito et al., 2006), the two clusters converge towards the medial axis and transiently merge under the trunk during early somitogenesis. However, the merged PGC clusters, thereafter, split to form two distinct clusters in mid-somitogenesis. In *G. holbrooki*, PGC cluster formation and their mobility resembles the former pattern. In most teleosts, following the localisation of two clusters at body mid-line, the PGCs are localised anterior to the putative gonadal ridge (PGR), thereafter, homing posteriorly reaching the PGR (Saito et al., 2006; Herpin et al., 2007; Herpin et al., 2008). In contrast, the first appearance of these two PGC clusters posterior to the PGR and their subsequent anterior migration towards PGR, in *G. holbrooki*, appears a developmental novelty in teleosts. Interestingly, this resembles the pattern of PGC migration in mouse, where they are first specified in the posterior primitive streak, move to subjacent endoderm and migrate anteriorly towards gonadal ridge (Anderson et al., 2000; Saitou and Yamaji, 2012). During somitogenesis, the route of these PGC cluster migration in some teleosts, *e.g.,* medaka, loach and goldfish (Saito et al., 2006), are longer compared to those in zebrafish (Doitsidou et al., 2002), gobby and herring (Saito et al., 2006). In *G. holbrooki*, this migration from the posterior body cylinder to the PGR (*i.e.,* homing at 4–7 somites) appears even longer than medaka (10–12 somites) and loach (15–17 somites). This cellular remodelling (*i.e.,* migratory direction and relatively long migration path) may be attributed to the reproductive adaptation from lecithotrophy of egg layers to that of live-bearing. Conceivably, the organogenesis machinery directs the gonial stem cells to the far anterior part of the body in live-bearing fish to co-accommodate the complex anatomical novelty of a fused ovary and proto-uterus such as in *G. holbrooki*. This anterior location is perhaps reset more medially in mouse with the anatomical distinction of ovary and uterus. A comparative evaluation encompassing multiple poeciliids with increasing degree of matrotrophy will no doubt shed greater light on evolutionary relatedness of PGC development and lecitho-matrotrophy.

### Only *Vasa* and *Dnd-α* Exclusively Mark PGCs in *G. holbrooki*

The exclusive occurrence of *vasa* and *dnd-α* in PGCs of *G. holbrooki* is comparable to *vasa* (Yoon et al., 1997; Knaut et al., 2000; Su et al., 2014) and *dnd* (Hong et al., 2016; Wargelius et al., 2016) of other teleosts and those *vasa* (Tanaka et al., 2000) and *dnd-α* (Youngren et al., 2005) of mice. Inferred from its maternal inheritance and exclusive detection in migrating PGCs, *vasa* likely underpins PGC formation and function in *G. holbrooki*. This is consistent across taxa with maternally inherited mode of germline determination (Styhler et al., 1998; Kuznicki et al., 2000; Tanaka et al., 2000; Hickford et al., 2011). Functionally, the DEAD-box domain of VASA proteins underpin regulatory roles which restructure and remodel protein-coding RNAs to facilitate their translation (Linder, 2006; Sengoku et al., 2006). Thus, maternal *vasa* is likely to facilitate the translation of Gp markers (predominantly stored in RNA form) in early zygotes of gambusia, as also occurs in lecithotrophic teleosts such as zebrafish (Knaut et al., 2002).

The abundant expression of maternal *dnd-α* in ova is likely associated with its essential role for specification and maintenance of PGCs in teleosts (Liu and Collodi, 2010; Hong et al., 2016). However, its established role as an anchor for maternal RNAs in unfertilised eggs and polymerization of cortical microtubule arrays has been demonstrated in *Xenopus* (Mei et al., 2013) needs verification in teleost. The subsequent occurrence of *dnd* in the embryonic and postnatal germline of *G. holbrooki* is consistent with those reported for other vertebrates (Youngren et al., 2005; Aramaki et al., 2007) including teleosts (Su et al., 2014; Duan et al., 2015; Wargelius et al., 2016) maintaining germline identity by protecting transcripts from miRNA-mediated decay (Ketting, 2007; Goudarzi et al., 2013), facilitating translation of essential germ plasm components for PGC development (Aguero et al., 2017), preventing PGCs from soma differentiation (Weidinger et al., 2003; Gross-Thebing et al., 2017) and titrating the abundant mRNA through transcription silencing (Nousch et al., 2013; Yamaji et al., 2017). Taken together, the exclusive expression of *dnd-α* in gambusia germ cells encoding the essential regulatory domains suggests a conserved role for it in germline maintenance in this species and likely in other poeciliids.

### Somatic Expression of Germline Markers May Confer Their Stemness

The non-germ cell-specific expression of *dazl*, *piwiII,* and *dnd-β*, in *G. holbrooki* is in accordance with the broader expression domain of these markers in invertebrates and higher vertebrates. For example, *Piwi* in invertebrates is also expressed in soma. Hence, it’s a suggested role beyond germline maintenance, assisting in somatic cell cycles (Ma et al., 2014), tissue regeneration and homeostasis (Palakodeti et al., 2008), adaptive immunity (Miesen et al., 2015) and sex determination (Kiuchi et al., 2014). Also, the expression domain of *piwi* in PGC and CNS of *G. holbrooki* is in agreement with those of zebrafish (Tan et al., 2002), medaka (Li et al., 2012), and mammals (Kim, 2019).

This study, for the first time, reports the presence of an alternately spliced variant of *dnd* (*dnd-β*) in any teleost during its embryonic development. Interestingly, the somatic expression of *dnd-β* mostly resembles those reported (Bhattacharya et al., 2007) in pre-natal stages of mice. However, the content and expression domain of the variants in mice (Youngren et al., 2005) and gambusia are different. Specifically, in mice, the larger variant (*Dnd-α*) is detected in prenatal stages, both in PGCs and soma (*e.g.,* neuroectoderm, head mesenchyme, neural tube, and hindgut), while the shorter variant (*dnd-β*) takes over post-natal function in a germline-specific manner (Bhattacharya et al., 2008). Whereas in *G. holbrooki*, both variants are expressed in prenatal embryos albeit in different domains, with the shorter *dnd-β* retaining germ cell-specific expression as in mice.

More broadly, the study presents evidence for an expansion (*dnd-β*, *dazl*) in expression domains of germ cell-specific markers to soma in *G. holbrooki*. Indeed, these genes encode RBPs that facilitate cell differentiation and maintenance of early cell lineages in vertebrates (Guallar and Wang, 2014; Shigunov and Dallagiovanna, 2015) and invertebrates (Kerner et al., 2011) via silencing, protecting and/or enhancing translation. (Tsui et al., 2000), (Xu et al., 2013). Similarly, in *G. holbrooki*, the expression of germline-RBP genes in CNS represents an evolutionary diversification of function, likely associated with maintaining somatic stemness and its renewal (Isler, 2013; Tsuboi et al., 2018).

## Conclusion

As a result of this study, our understanding of germ cell development in poeciliids has been markedly enhanced yielding insights shared with lecithotrophic teleosts as well as matrotrophic mammals. Specifically, the PGC-marker genes allowed the mode of PGC sequestration and position of germline to be determined. Remarkably, *G. holbrooki* retained preformation mode (*i.e.,* maternal inheritance) of specification (like teleosts) with a relatively early zygotic activation. Also, the early spatial pattern of PGCs resembled that of zebrafish, while the later migration pattern reminiscent of medaka and mouse, sharing the features of both lecithotrophy and matrotrophy. Consistent with this adaptation, the expression of teleostean germ cell markers in somatic cells confers stemness of the latter. Regardless, it is yet unclear if the shift in these molecular and cellular patterns led to evolution of the reproductive strategies or vice versa. In part, the adaptations in spatial patterns could be related to geometric restriction on developing embryo in matrotrophy compared to those of lecithotrophic teleosts, an aspect that needs verification. Showing the previously unsuspected conservation of elements specific to both lecithotrophic and matrotrophic organisms in *G. holbrooki*, lends itself as an excellent system to understand the evolutionary connectedness of germline development modes, reproductive adaptations and functional diversity of the germline markers. Like zebrafish, *G. holbrooki* is easy and relatively inexpensive to maintain, but display greater similarity to higher vertebrates, making it far more attractive system to study and understand mechanism of reproductive development and associated disease conditions of higher vertebrates. Also, a knowledge of PGC markers and functions is likely to provide pathways to develop species-specific solution for managing pest populations of this invasive species.

## Data Availability

The datasets presented in this study can be found in online repositories. The names of the repository/repositories and accession number(s) can be found below: NCBI [accession: MZ542286-MZ542293].
