# Supplementary material for: Primordial Germ Cell Development in the Poeciliid, Gambusia holbrooki, Reveals Shared Features Between Lecithotrophs and Matrotrophs
Source: Front Cell Dev Biol. 2022 Mar 1;10:793498. doi: 10.3389/fcell.2022.793498 (PMC8920993; doi:10.3389/fcell.2022.793498)
Supplement: Supplementary file 1 [file Table1.DOCX]

**Supplementary Table S1. Housing condition of *Gambusia* experiment**

| **Parameters** |  | **Range** |
| --- | --- | --- |
| **Water quality** | Temperature (˚C) | 25.0 ±1.0 |
|  | pH | 7.1-7.5 |
|  | Dissolved oxygen (%) | 85-90 |
|  | Salinity (g/l) | 0 |
|  | NO**_2_** (mg/l) | <0.008 |
|  | NH**_4_^+^** (mg/l) | <0.009 |
| **Rearing conditions** | Female tank volume (l) | 6 |
|  | male tank volume (l) | 2 |
|  | Water flow (l/h) | 10-13 |
|  | density (fish/10 L tank) | 15-25 |

**Supplementary Table S2. Oligomers used in qPCR and for generating wholemount ISH probes.**

| **Gene** | **Accession number** | **Primer name** | **Sequence (5′– 3′)** | **Length (bp)** | **Purpose** |
| --- | --- | --- | --- | --- | --- |
| *vasa* | MZ542293 | ghvasa170Fq3 | GCAAAGGGAGAGGCAGAGGAGGA | 108 | q-PCR |
|  |  | ghvasa277Rq3 | CTCTACCGCCTCTCCCACTGAAACCA |  |  |
|  |  | ghvasa36Fp | CATAAATATGGACGAGTGGGAAGAAG | 1122 | WMISH |
|  |  | ghvasa-79Rp | ATGTCCAACATCCGGTCGGCCTCGTC |  |  |
| *nanos1* | MZ542291 | ghnos503Fq2(P) | TCGATCTCTTTGGTGTGGAGCGGAAGAT | 91 | q-PCR |
|  |  | ghnos93Rq2 | CCGTTATTCCGGCAGAACACGCAGATTT |  |  |
|  |  | ghnos503Fq2(P) | TCGATCTCTTTGGTGTGGAGCGGAAGAT | 716 | WM-ISH |
|  |  | ghnos1-1218Rp | ACATAACTTTGGTTTCTCTAAGACATG |  |  |
| *dnd-α* | MZ542289 |  | CTCCAGCCCCAAAGATTCTGCT | 99 | q-PCR |
|  |  |  | CCCTGCAGAACCCTGGAGAGA |  |  |
|  |  | ghdnd68Fp | GCCTGGCTGGAAATGACCAA | 896 | WM-ISH |
|  |  | ghdnd963Rq2(p) | CCATCCGGTCTGGCCAAACTGTAGTG |  |  |
| *dnd-β* | MZ542290 | ghdnd102F(q) | GTTAACGGGCAGAGGAAGTACGGAGGA | 96 | q-PCR |
|  |  | ghdnd66Rq3 | CCGAGGGATGTGGCTGATGAAGACC |  |  |
|  |  | ghdnd68Fp | GCCTGGCTGGAAATGACCAA | 830 | WM-ISH |
|  |  | ghdnd963Rq2(p) | CCATCCGGTCTGGCCAAACTGTAGTG |  |  |
| *piwi II* | MZ542292 | ghpiwi42Fq3(p) | TGAACACCTGGTGAGACGGATCGGAA | 103 | q-PCR |
|  |  | ghpiwi144Rq3 | TGGAAGTCTGCCTTGTCACTGAGGTCA |  |  |
|  |  | ghpiwi42Fq3(p) | TGAACACCTGGTGAGACGGATCGGAA | 1419 | WM-ISH |
|  |  | ghpiwi1460Rq2(p) | ACAGCCCTCCATCTGTGCGCTTAATA |  |  |
| *dazl* | MZ542288 | ghdazl303Fq4 | GGGTTTGCAAAGGGTACGGGTTTGTGT | 104 | q-PCR |
|  |  | ghdazl406Rq3 | GCCCAGCTTGAGTTTCCGCCCTTTA |  |  |
|  |  | ghdazl1F231 | AGGGCAGACTGACCCCCAA | 908 | WM-ISH |
|  |  | ghdaz1058Rp | TGGCTGGTATACCAAATGTGCTCT |  |  |
| *Β-actin* | MZ542286 | ghBact46Fq3 | CGGCAGGACTTCACCTACAGACACCT | 99 | qPCR |
|  |  | ghBact144Rq3 | CTTGCACAAACCGGAGCCGTTGTCA |  |  |
